# Field-induced orientational switching produces vertically aligned Ti_3_C_2_T_*x*_ MXene nanosheets

**DOI:** 10.1038/s41467-022-33337-2

**Published:** 2022-09-24

**Authors:** Changjae Lee, Soon Mo Park, Soobin Kim, Yun-Seok Choi, Geonhyeong Park, Yun Chan Kang, Chong Min Koo, Seon Joon Kim, Dong Ki Yoon

**Affiliations:** 1 Department of Chemistry, Korea Advanced Institute of Science and Technology, Daejeon, 34141 Republic of Korea; 2 Graduate School of Nanoscience and Technology, Korea Advanced Institute of Science and Technology, Daejeon, 34141 Republic of Korea; 3 Materials Architecturing Research Center, Korea Institute of Science and Technology, 5, Hwarang-ro 14-gil, Seongbuk-gu, Seoul, 02792 Republic of Korea; 4 Department of Materials Science and Engineering, Korea University, 145, Anam-ro, Seongbuk-gu, Seoul, 02841 Republic of Korea; 5 Division of Nanoscience and Technology, KIST School, University of Science and Technology, 5, Hwarang-ro 14-gil, Seongbuk-gu, Seoul, 02792 Republic of Korea; 6 School of Advanced Materials Science and Engineering, Sungkyunkwan University, Suwon, 16419 Republic of Korea; 7 Convergence Research Center for Solutions to Electromagnetic Interference in Future-mobility, Korea Institute of Science and Technology, 5, Hwarang-ro 14-gil, Seongbuk-gu, Seoul, 02792 Republic of Korea

**Keywords:** Molecular self-assembly, Self-assembly, Colloids, Self-assembly, Liquid crystals

## Abstract

Controlling the orientation of two-dimensional materials is essential to optimize or tune their functional properties. In particular, aligning MXene, a two-dimensional carbide and/or nitride material, has recently received much attention due to its high conductivity and high-density surface functional group properties that can easily vary based on its arranged directions. However, erecting 2D materials vertically can be challenging, given their thinness of few nanometres. Here, vertical alignment of Ti_3_C_2_T_*x*_ MXene sheets is achieved by applying an in-plane electric field, which is directly observed using polarised optical microscopy and scanning electron microscopy. The electric field-induced vertical alignment parallel to the applied alternating-current field is demonstrated to be reversible in the absence of a field, back to a random orientation distribution. Interdigitated electrodes with uniaxially aligned MXene nanosheets are demonstrated. These can be further modulated to achieve various patterns using diversified electrode substrates. Anisotropic electrical conductivity is also observed in the uniaxially aligned MXene nanosheet film, which is quite different from the randomly oriented ones. The proposed orientation-controlling technique demonstrates potential for many applications including sensors, membranes, polarisers, and general energy applications.

## Abstract (web summary)

In this work, authors demonstrate reversible vertical alignment of Ti_3_C_2_T_x_ MXene sheets induced by an applied in-plane electric field. Further modulation of the field can achieve programmed patterns onto various electrode substrates.

## Introduction

MXenes, a family of two-dimensional (2D) transition metal carbides, nitrides, and carbonitrides with a chemical formula of M_n+1_X_n_T_*x*_ (M = early transition metals, X = carbon and/or nitrogen, T_*x*_ = functional groups, *n* = integer between 1 and 4), are one of the most intensively studied two-dimensional (2D) materials in recent days^1–3^. Their exceptional electrical conductivity, variable surface functional groups^4^, and dispersibility^5,6^ in a wide range of solvents have been exploited in delivering world-best properties in applications such as supercapacitors^7–9^, electromagnetic interference shielding^10–13^, photovoltaics^14^ and sensors^15–18^. Additionally, above a critical concentration, the MXene dispersion exhibits the lyotropic liquid crystal (LC) phase due to the high aspect ratio of flakes with micrometre-scale lateral dimensions and a few nm thickness^19,20^. Based on Onsagar’s theorem for 2D materials^21–23^, the excluded volume of the MXene flakes develops the ordered phase spontaneously, where the long-range orientational order of the flakes can be controlled by using external stimuli. Desired functionalities of MXene can be enhanced by control of the structural anisotropy, which brings it one step closer to practical applications like other liquid crystalline materials^24–26^. However, to the best of our knowledge, only a few studies have addressed the interplay of order and orientation of MXene sheets. It is because aligning 2D MXene sheets, especially in a vertical manner on a substrate, is challenging due to the flexibility of sheets^27,28^ and entropic penalty due to gravitational force^20^.

For example, Xia et al. introduced non-ionic surfactant molecules to MXene sheets for enhancing cofacial assembly to fabricate a vertically aligned lamellar structure via mechanical shearing^7^. This scalable approach realised a supercapacitor with thickness-independent performance with high rate capability^7^. Other studies have found that template-assisted self-assembly, such as entwined metal mesh^29^ and PDMS rectangular channels^27^, can induce the vertical alignment of MXene sheets to reveal improved electrochemical properties. However, the previous studies need additional efforts to form a double layer of liquid crystalline surfactant on the colloid surface, and it is hard to get the global alignment in a large area. Moreover, there have been no reports on the active and reversible control of the alignment of pure MXene nanosheets into the designated patterns.

Here, we have rationalised a system that can control the orientation of MXene sheets and modulate their electro-optical characteristics. The well-designed electrodes are used to apply an in-plane electric field to MXene sheets and actively switch their orientation. The resulting highly ordered and oriented MXene sheets exhibit linear dichroism in the visible light range. The degree of alignment varies depending on the voltage and spacing between the electrodes, which is directly investigated using in-situ polarised optical microscopy (POM) and scanning electron microscopy (SEM). The conductivity of the MXene film is measured using the two-terminal method to demonstrate the alignment-dependency of electrical properties. Based on this platform, the ordered two-dimensional MXene structure can be built into designated patterns on an arbitrary scale by changing the layout of electrodes. We believe that our facile method to control the orientation of MXene sheets can give insight into utilizing ordered MXene structures in potential applications.

## Results

### Vertical alignment of MXene nanosheets under an AC electric field

**Fig. 1:**
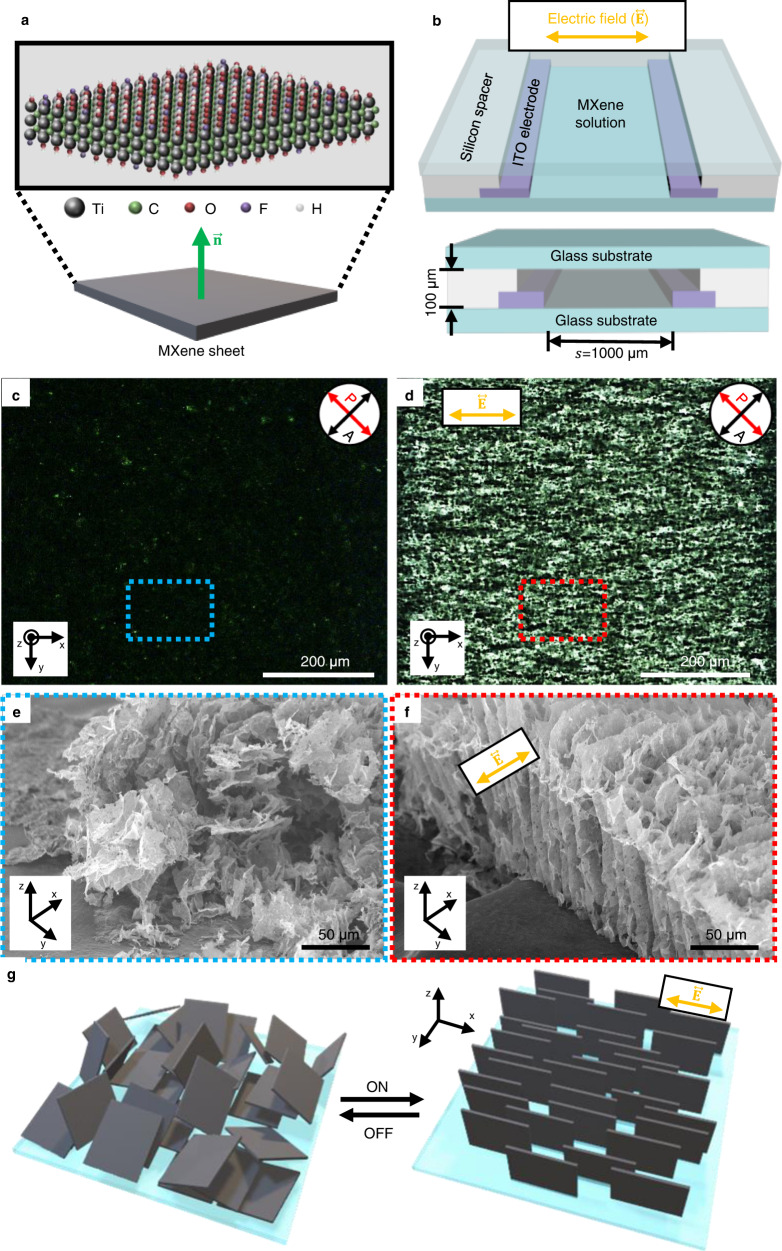
Electric field-induced alignment of MXene sheets. **a** Atomic structure of MXene nanosheet and its schematic illustration. $\vec{{{{{{\bf{n}}}}}}}$ is normal verctor of a MXene sheet. **b** Schematic illustration of electrode-patterned cell. $s$ is separation between the electrodes. **c**, **d** POM images of MXene samples without (**c**) and with (**d**) an AC electric field application. P and A symbols represent the polariser and analyser, respectively. **e**, **f** SEM images freeze-dried MXene nanosheets prepared under no electric fi**e**ld (**e**) and under an electric field (**f**). **g** Schematic illustration of reversible modulation of MXene nanosheet orientation in the condition of ON or OFF states of the electric field.

Ti_3_C_2_T_*x*_ MXene is synthesized by etching Ti_3_AlC_2_ MAX phase with a LiF/HCl etchant (details in the Experimental Section), which typically yields delaminated MXene sheets with average lateral sizes of 2~3 μm, as shown in the SEM and TEM images shown in Supplementary Fig. S1. In addition, zeta-potential results show the negatively charged surface of individual MXene sheets, and the pH value of the MXene aqueous solution was near 6. Completely delaminated MXene sheets have a high density of oxygen, hydroxyl, and fluorine functional groups on the surface (Fig. 1a), resulting in a homogeneous, aqueous colloidal solution. The concentration of MXene solution is tuned to 10 mg/ml, which is slightly above the critical concentration to form the LC phase^19^, and injected into a closed cell as illustrated in Fig. 1b. Parallel indium tin oxide (ITO) electrodes are designed and fabricated with a line width (*w*) of 500 μm and 1000 μm separation (*s*) to apply in-plane electric field ($\vec{{{{{{\bf{E}}}}}}}$) on the MXene solution (Supplementary Fig. S2 in Supporting Information). A typical slide glass covers the solution to block water evaporation, where a silicon rubber spacer is used to fix the gap at 100 μm.

When the MXene solution is observed under POM shortly after sample preparation, bright spots are scattered across the dark background, showing the isotropic-nematic biphase^7,19^ (Fig. 1c). However, the optical texture becomes different in the presence of an alternating current (AC) electric field (Fig. 1d). The applied AC field is $V={V}_{0}{{\sin }}2\pi {ft}$, where ${V}_{0}$ and $f$ are the voltage amplitude and frequency, respectively. Here, the ${V}_{0}$ sets to 20 V at a high frequency of 10 kHz. The applied field is kept for 60 s. The bright domains in POM images indicate a change in the optical birefringence, meaning that the electric field can modulate the orientation of MXene sheets. This phenomenon is caused by structural anisotropy and significant surface charge^30^ (Supplementary Fig. S1d) of MXene nanosheets. An electric field application aligns anisotropic particles in colloidal dispersion such as metal organic framework^31,32^, carbon nanotube^33–35^ and graphene substituents^36,37^, because strong dipole moment is induced by solvent and counterion cluster at the interface of colloids. It strengthens the dielectric and conductive characteristics of anisotropic colloids, and consequently, a torque is applied to the particle for aligning parallel to the electric field. In the MXene colloidal solution, the structurally anisotropic ion cloud around the nanosheet surface induces a large polarisation parallel to the applied electric field with a high frequency^38^. Also, the highly charged surface results in cofacial and vertical alignment under the electric field to maximise electrostatic repulsion^39–41^.

Also, the field-induced orientational change is investigated depending on the cell thickness (Supplementary Fig. S3a, b). The thickness of 100 µm shows a much larger increase in birefringence intensity than the thinner cases, from which the designed cell thickness is determined to be 100 µm. It could be argued that sufficient space is required for the MXene sheets to be aligned along the applied electric field. To support this more clearly, the behaviour of a large particle with the lateral size of ~10 µm is also observed by POM under the electric field (Supplementary Fig. S3c). Rotation and dislocation don’t occur even at the voltage due to a lack of freedom for the large particle, although the birefringence becomes slightly increased at the particle domain due to the dynamic scattering for 2D liquid crystalline materials^42^.

The alignment state of MXene sheets is further examined to see the detailed internal structure using SEM (Fig. 1e, f), where MXene samples prepared with/without electric field were freeze-dried to fix the sheet orientation. In the sample prepared without an electric field, MXene sheets are randomly aligned on the substrate, i.e., a mixture of parallel, perpendicular, and tilted sheets to the bottom substrate (Fig. 1e and Supplementary Fig. S4a), which well-matches with the results indicated by the POM image (Fig. 1c). On the contrary, the sample aligned under the electric field shows vertically oriented MXene sheets to the bottom substrate, in which layer normal direction ($\vec{{{{{{\bf{n}}}}}}}$) of the sheets is aligned perpendicular to $\vec{{{{{{\bf{E}}}}}}}$ (Fig. 1f and Supplementary Fig. S4b). The resultant MXene structures directly show that the electric field effectively modulates the nematic ordering from random to aligned state, as illustrated in Fig. 1g, revealing the reconfigurable MXene orientation.

### Optical analysis of aligned MXene nanosheets

**Fig. 2:**
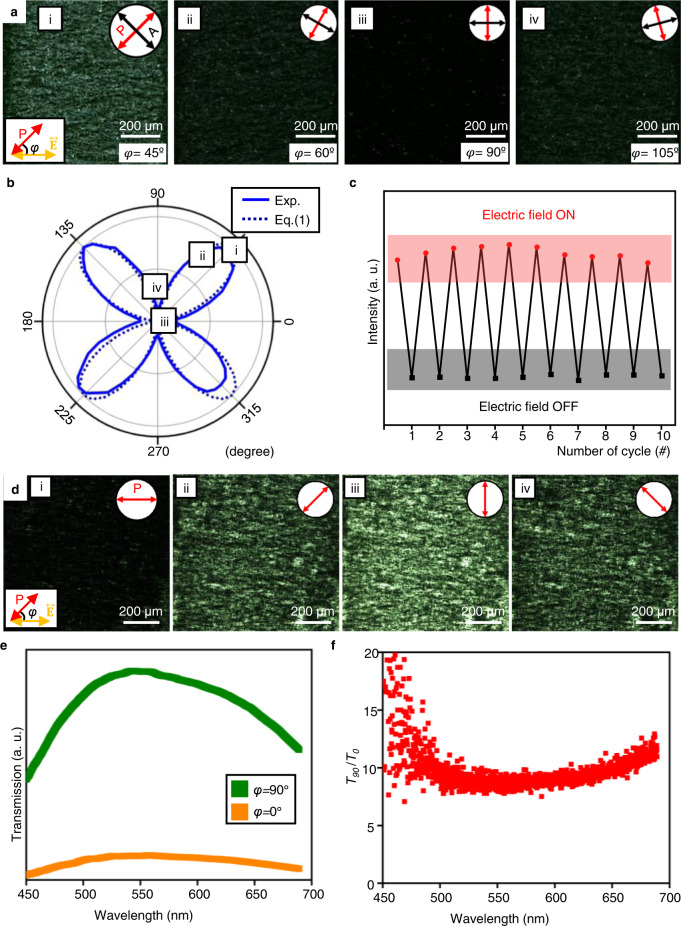
Optical properties of MXene nanosheets aligned under an AC electric field. **a** POM images of the aligned MXene nanosheets with rotating the crossed polarisers. $\varphi$ is the angle between the polariser and the applied electric field. **b** Polar plots depending on the rotating angle of the crossed polarisers. The solid line is the experimental value and the dotted line is the theoretical value plot. **c** Cyclic birefringent intensity change. The red circles and black squares indicate the birefringence intensity when the electric field is on and off, respectively. **d** OM images of the aligned MXene nanosheets with rotating a single polariser. **e** Transmission of aligned MXene nanosheets with two different polariser angles of 90° and 0°. **f** Ratio between the transmissions at the polariser angles of 90° (*T*_*90*_) and 0° (*T*_*0*_). Source data are provided as a Source Data file.

The optical characteristics of the oriented MXene sheets are directly investigated to trace the birefringence changes and linear dichroism upon rotating polarisers (Fig. 2). First, a series of POM images according to rotating cross-polarisers show the distinctive birefringence changes of the highly aligned MXene sheets under an AC electric field (Fig. 2a). Here, $\varphi$ is defined as the angle between $\vec{{{{{{\bf{E}}}}}}}$ and polariser (P) vector, which is varied (Fig. 2a). a-i) $\varphi$ = 45°: The domain between the electrodes seems the brightest and has a consistent texture, as shown in Fig. 1d. a-ii) $\varphi$ = 60°: Optical textures look similar, but the brightness decreases by 25%. a-iii) $\varphi$= 90°: As the angle reaches 90 degrees, the image becomes dark, indicating the aligned MXene sheets along with the polariser or analyser. a-iv) $\varphi$= 105°: The domain gets brighter again. The resulting intensities are quantified by rotating cross-polarisers at intervals of 5 degrees from 0 to 360 degrees, revealing the polar plot of angular dependence (Fig. 2b). The polar plot shows a 4-fold symmetry, in which the highest transmittance is shown at $\varphi$= 45° + 90° * n (n is an integer), while the lowest transmittance is detected at $\varphi$= 90° * n. It matches with the theoretical values of birefringent intensity of uniaxially aligned materials, which is described by Eq. (1):

1 $$I\propto {I}_{0}{{{\sin }}}^{2}2\varphi$$

where $I$ is the transmitted light intensity and ${I}_{0}$ is the incident light intensity. Reversible switching is demonstrated by comparing the birefringent intensity of the POM images while turning the electric field on and off repeatedly 10 times (Fig. 2c). As soon as an electric field is applied, the MXene nanosheet is aligned, and the birefringence intensity increases immediately. When turning off the electric field, the nanosheets alignment is wholly relaxed and returns to the initial state after about 60 s. (Supplementary Fig. S5) The iterative change of the POM image is recorded as a video provided in Supplementary Movie 1.

The dichroic characteristics of aligned MXene are also observed in the same experimental condition. We observe the aligned sample through OM with a single polariser inserted. The brightness of OM images is gradually changed, in which the absorption of the polarised light is maximised at $\varphi$= 0° and minimised at $\varphi$= 90° (Fig. 2d i–iv). From this result, an anisotropic interaction is explicitly shown between MXene sheets and linearly polarised light in the visible range. The transmitted intensity is measured for Figs. 2d-i and 2d-iii in the visible light range to examine the degree of linear dichroism, where *T*_*0*_ and *T*_*90*_ are the transmittances at $\varphi$= 0° and 90° (Fig. 2e). Both graphs show the convex curves with the maximum value near ~540 nm, which is in line with the absorption spectrum of Ti_3_C_2_T_*x*_ MXene (Fig. 2d)^43^. *T*_*90*_ is much higher than *T*_*0*_ in the measured range and the ratio *T*_*90*_ /*T*_*0*_ is used to estimate the degree of linear dichroism (Fig. 2f). On average, ${T}_{90}$ is about 10 times higher than ${T}_{0}$, and the ratio gets higher in short-wavelength due to the enhanced absorption of the aligned MXene sheets^44^. It shows the aligned MXene sheets in our platform can be used in the applications such as smart windows, polarisers, and compensation film.

The resultant MXene sheets aligned parallel to the applied AC electric field are quite different from the previous work showing the perpendicular orientation to the direct current (DC) field (Supplementary Fig. S7)^45^. To compare the difference between the two current modes in our study, we also analyse the optical characteristics of MXene sheets under the DC electric field. The OM image of the sample has a uniform brightness in the initial state (Supplementary Fig. S7a). However, the local brightness gradually changes to show a brightness gradient under DC field, where the anode region darkens while the cathode region becomes oversaturated (Supplementary Fig. S7b–e). It may result from the migration of the charged MXene sheets from cathode to anode, which can be explained by the electrophoretic effect^45^. Even though water electrolysis is observed as formation of bubbles near the electrode under a DC field, it doesn’t have significant effect on the MXene migration, because the deposition process is completed within a few seconds. Therefore, the utilization of AC mode is essential to generate stable and uniformly aligned MXene sheet architectures.

### Optical textures in different voltages and electrode separations

**Fig. 3:**
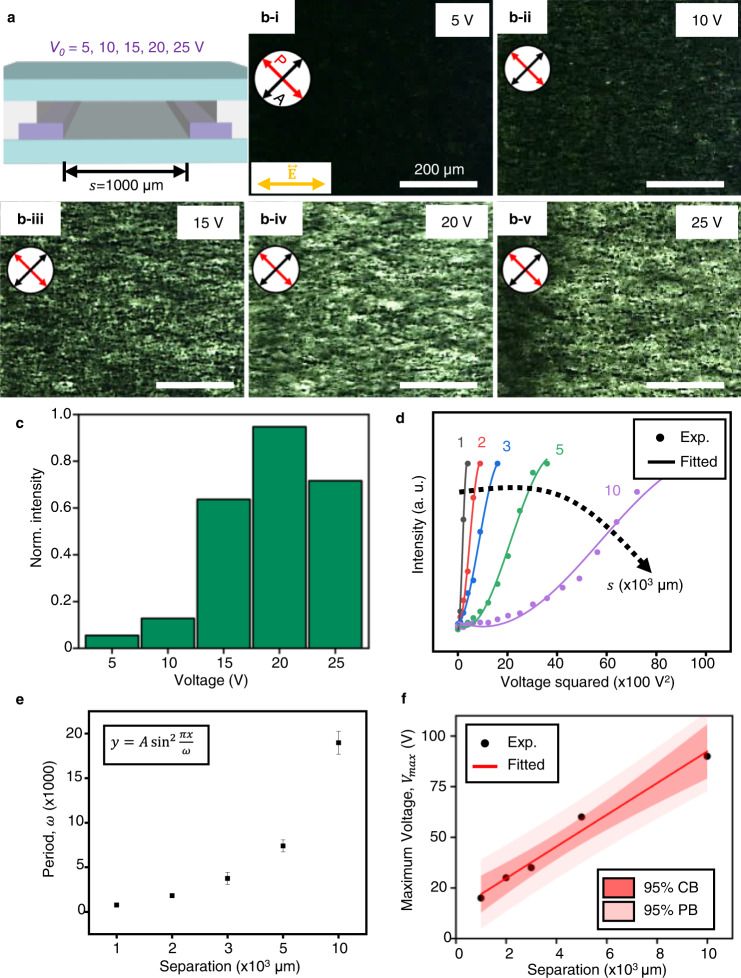
Voltage- and Electrode separation-dependent optical texture analysis of MXene nanosheets. **a** Schematic illustration of cell to show the variables. ${V}_{0}$ is the applied voltage of an AC electric field. **b** POM images with different brightness depending on the voltage. **c** Normalised birefringent intensity varying the applied voltage with fixed an electrode separation of 1000 μm. **d** Fitted graph of birefringent intensity of different electrode separation depending on the applied voltage squared. Dots and solid lines are the experimental results and fitted plot, respectively. **e** Period (ω) of the fitted sine-squared plots depending on the electrode separation. **f** The voltage producing the maximum intensity of birefringence (Maximum voltage, ${V}_{{\max }}$) for each electrode separation. CB and PB are confidence band and prediction band, respectively. Source data are provided as a Source Data file.

To investigate the factors that influence the alignment of the MXene sheets, we vary the voltage and width of the line electrodes (Fig. 3 and Supplementary Fig. S8). First, the birefringence change of the MXene is observed as ${V}_{0}$ is increased at a fixed electrode configuration with $s=$ 1000 μm (Fig. 3a). A series of POM images are captured at ${V}_{0}$= 5~25 V (Fig. 3b), in which the intensity of the birefringence is measured (Fig. 3c). Each voltage condition is applied for 60 s. Considering the brightness intensity for each voltage, the intensity rises until ${V}_{0}$= 20 V, then drops at ${V}_{0}$= 25 V. Especially, the brightness increases rapidly rather than linearly according to the voltage, which suddenly jumps by nearly four times when the voltage increases from ${V}_{0}$= 10 V to ${V}_{0}$= 20 V, indicating that the transition occurs from the isotropic to the ordered state under a particular electric field condition. And the nematic ordering of MXene sheets surges in response to the electric field. Although the strength of the birefringence typically rises as the electric field increases, the electrothermal effect should also be considered at a high voltage to explain the drop^46^.

The electrode separation varies from 1000 μm to 10000 μm, and the voltage variation experiments are also performed at each separation distance (Fig. 3d and Supplementary Fig. S8a). POM images are arranged as a function of $s$ and ${V}_{0}$, and the birefringence levels are also measured (Supplementary Fig. S8b–e). In all cases, the birefringence levels shift similarly to $s$= 1000 μm, with the value non-linearly increasing and peaking at a specific voltage. When we define ${V}_{{\max }}$ as the voltage which induces the maximum intensity of birefringence, ${V}_{{\max }}$ becomes higher as $s$ increases. Based on the experimental data trend available to five cases, we find the two correlations of birefringence with applied voltage and electrode distance. i) Non-linear increase of birefringence intensity: For all samples, we organise the measured brightness from 0 to ${V}_{{\max }}$ and fit the voltage-dependent intensity with a sine-squared function expressed as $y=$ $A \, {{{\sin }}}^{2}\frac{\pi x}{\omega }$, where $A$ is the amplitude, $\omega$ is the period, and x value is voltage squared (${V}_{0}^{2}$). The relationship between the birefringence intensity and applied electric field was reported^47–49^, which can be described as:

2 $$I\propto {{{\sin }}}^{2}(\pi {Ks}{E^{\prime} }^{2})$$

where ${I}$, $K$ and $E^{\prime}$ are birefringence intensity, Kerr coefficient and applied electric field, respectively. Based on Eq. (2), the sine squared function was determined to be a fitting function. As shown in Fig. 3d, the sine-squared function fits well with the rapid increase of birefringence with respect to the voltage changes at different *s* values, and the slope gets slower as $s$ increases (Fig. 3d), which directly shows the relationship between birefringence intensity and *s*. In other words, as *s* increases, the period ($\omega$) tends to increase (Fig. 3e). In our system, the electric field is generated between two coated electrodes, where the centre is similar to the parallel metal plates (${E}^{{\prime} }=V/s$)^50^. Therefore, as $s$ increases, more voltage is required. ii) For each $s$, we yield ${V}_{{\max }}$ and find a linear relationship (Fig. 3f). The scattered points are covered by 95% confidence band (CB), which represents 5% of uncertainty in an estimate of the function based on noisy data. And 95% prediction band (PB), which represents the uncertainty about a new data-point subject to noise, indicates a linear growth. Through these optimization processes, the ${V}_{{\max }}$ for each interval can be predicted within a specific range.

### Complex structure modulation of vertical MXene nanosheets

**Fig. 4:**
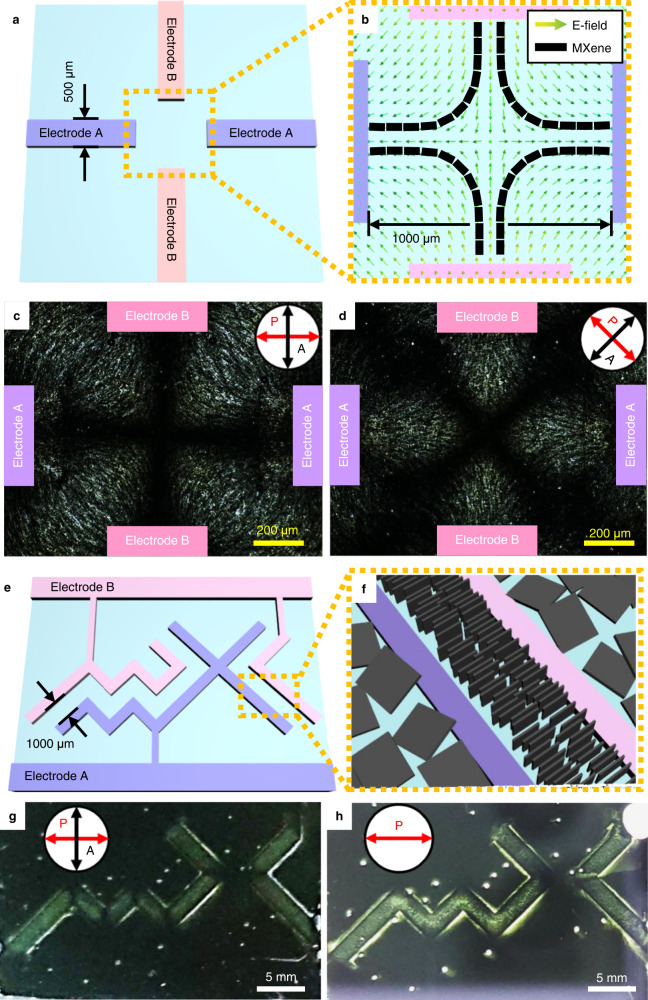
Two-dimensional structure of aligned MXene sheets via electrode design. **a** Schematic illustration of windmill-like electrode pattern. **b** Electric field vector and corresponding aligned vertical MXene nanosheets in the middle of the electrode pattern. The yellow-green gradient arrows indicate the direction of the applied electric field. The black bars represent the orientation of each vertically aligned MXene nanosheet. **c**, **d** POM images of aligned MXene nanosheets showing Maltese cross pattern. **e** Schematic illustration of more complicated electrode pattern of “MX”. The spacing between the electrodes forming the pattern is 1000 µm. **f** Structure of aligned MXene nanosheets in the inter-electrode area of the “MX” pattern. **g**, **h** Optical images of aligned MXene nanosheets showing bright “MX” alphabets.

Beyond a simple one-dimensional arrangement, we design electrodes that can create a two-dimensional electric field. Four electrodes are located at the cardinal points with an empty square centre, and each facing set of electrodes, with a 1000 μm distance, are assigned as Electrodes A and B (Fig. 4a). When a signal is applied to Electrode A and ground to Electrode B, a hyperbolic electric field is generated in the centre area, in which the MXene sheet orientation can be visualised by simulation (Fig. 4b), using the fact that $\vec{{{{{{\bf{n}}}}}}}$ is perpendicular to $\vec{{{{{{\bf{E}}}}}}}$. In the presence of the electric field, MXene sheets display a Maltese cross pattern with four-fold symmetry under POM (Fig. 4c), and the pattern rotates in lockstep with the spin of the cross-polariser (Fig. 4d). It indicates that vertically-aligned MXene sheets form the nematic LC texture with −1 defect, as illustrated in Fig. 4b.

More complicated in-plane electrodes are designed to extend the versatility of our platform, in which the electrodes were patterned to represent alphabets “MX” (Fig. 4e). Each letter stroke, which comprises two parallel electrodes separated by a 1000 µm distance, is drawn at 45 degrees with one of the crossed polarisers. (Fig. 4f). Due to the field-induced orientation of MXene sheets, the bright domain arises when an electric field is applied and matches the designed electrodes’ pattern (Fig. 4g). The area where the letter’s stroke is bent appears dark due to the parallel or perpendicular alignment of MXene in respect to polarisers. The OM image with the single polariser inserted exhibits similar phenomena due to the dichroic characteristics of MXene sheets (Fig. 4h). The original photos of Fig. 4g, h are provided in Supplementary Fig. S9. This result suggests that various configurations of MXene sheets can be achieved by designing the arrangement of the electrodes.

### Anisotropic electrical conductivity of aligned MXene film

**Fig. 5:**
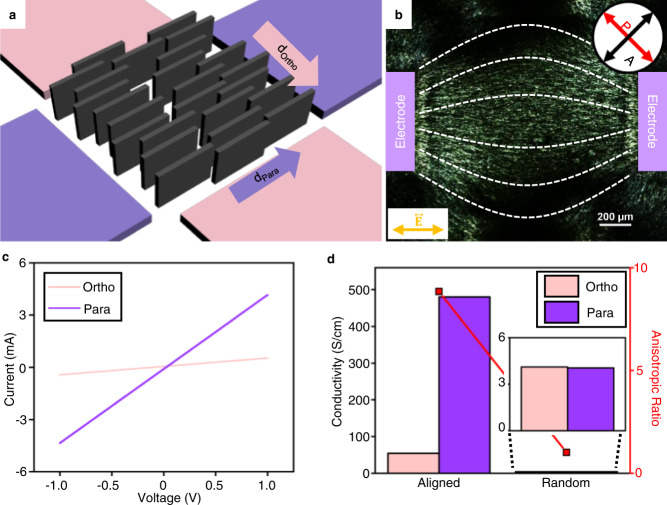
Alignment-dependent electronic conductivity of MXene nanosheet film. **a** Schematic illustration of aligned MXene nanosheet film in the windmill-like electrode pattern to have different electrical conductivities in directions parallel (${d}_{{para}}$) and orthogonal (${d}_{{ortho}}$) to the orientation. **b** POM image of aligned MXene sheet film to be freeze-dried to fix its alignment. **c** Current-voltage (I-V) curve in ${d}_{{para}}$ and ${d}_{{ortho}}$. **d** Alignment-dependent conductivities (${\sigma }_{{para}}$ and ${\sigma }_{{ortho}}$) and corresponding anisotropic ratio (${\sigma }_{{para}}/{\sigma }_{{ortho}}$) of aligned and randomly oriented samples. Source data are provided as a Source Data file.

We also try to demonstrate that the alignment of MXene sheets can induce anisotropy to materials’ functionalities. As the proof of concept, the electrical conductivity is measured depending on the alignment state. The same electrode set as Fig. 4a is used, but an electric field is only applied between a single pair (purple colour) of electrodes (Fig. 5a). As a result, a bright domain appears between the electrodes in the POM image (Fig. 5b), indicating $\vec{{{{{{\bf{n}}}}}}}$ of MXene sheets is aligned perpendicular to the electric field. This alignment is fixed by the freeze-drying method. The electrical conductivity measurement (I − V curve) of the resulting MXene structure is carried out by a two-terminal transport method. Each I-V curve is obtained in the range of unit voltage when the conductivity was measured between the electrodes located either parallel (${d}_{{para}}$) and orthogonal (${d}_{{ortho}}$) to the aligned MXene sheets (Fig. 5c). In both cases, the current of aligned MXene sheets increases linearly within a given voltage range to show an Ohmic behaviour. Also, MXene structures with randomly oriented sheets are fabricated without applying an electric field before freeze-drying to compare with the controlled case above. From the slope of the I-V curves, the conductivities (${\sigma }_{{para}}$, ${\sigma }_{{ortho}}$) of the aligned MXene sheets can be deduced and compared to the randomly oriented MXene sheets (Fig. 5d). First, the ${\sigma }_{{ortho}}$, and ${\sigma }_{{para}}$ of the randomly oriented sample are almost the same values of 4.11 S/cm and 4.04 S/cm, respectively, which is close to that of the reported aerogel fabricated with similar MXene concentration^51^. The two values are nearly identical due to the random alignment. In the case of aligned MXene sheets, the ${\sigma }_{{ortho}}$, and ${\sigma }_{{para}}$ are 54.85 S/cm and 480.23 S/cm, respectively. Interestingly, both ${\sigma }_{{para}}$ and ${\sigma }_{{ortho}}$ increase compared to those of the randomly aligned sample. This enhancement is explained by dielectrophoretic condensation that occurred under a strong electric field, as reported in the study of graphene oxide^52^, which dramatically increases the nematic packing density of the MXene solution. The dielectrophoretic condensation of MXene nanosheets is better shown in the solution in which the solvent is replaced with dimethyl sulfoxide (DMSO). (Supplementary Fig. S10). It should be noted that the sufficient inter-sheet space created by the larger molecular size of DMSO solvent makes it easier for the MXene nanosheets to be aligned along the electric field, resulting in much lower ${V}_{{\max }}$ (Supplementary Fig. S10a). Since the boiling point of DMSO is higher than that of water (189 °C), evaporation by electrothermal effect is suppressed even at high voltage. Therefore, enhanced dielectrophoretic condensation can be observed in DMSO solution, where concentration increases in the inter-electrode area at high voltages (Supplementary Fig. S10b). With continued condensation at increased voltages, the birefringence intensity keeps decreasing from its maximum as shown in Supplementary Fig. S10a. In addition, it is expected that the uniaxial alignment of the nanosheets further increased the packing efficiency and caused a synergistic effect^53^. Thus, a relatively high conductivity value can be demonstrated, despite the fact that the porous sample prepared by freeze-drying has a relatively low layer density. In addition, the aligned sample has anisotropic conductivity, where ${\sigma }_{{para}}$ is over 8 times higher than ${\sigma }_{{ortho}}$ because electron charge in the 2D MXene sheets favors to transport along the basal plane. In overall, a high anisotropic ratio (${\sigma }_{{para}}/{\sigma }_{{ortho}}$) of 8.83 is obtained in the aligned sample while the randomly oriented one has a low anisotropic ratio of 1.02 due to the nearly identical ${\sigma }_{{para}}$ and ${\sigma }_{{ortho}}$. Through this result, it can be suggested that the developed platform can endow anisotropy to MXene’s functionalities.

## Discussion

In summary, we implemented the reversible and large-area vertical alignment of MXene on the substrate by applying an in-plane AC electric field, in which MXene sheets are aligned parallel to the AC field. MXene sheets were vertically standing on the substrate, in which the vertical alignment could be directly observed through POM and SEM. By switching the electric field on and off, the alignment of the MXene sheets is reversibly modulated in our system along with their dichroic properties. Furthermore, we investigated the effect of applied voltage and electrode separation on the alignment of MXene sheets and deduced a correlation to estimate the optimum voltage to align MXene sheets for a given electrode separation. Complex patterns containing aligned MXene sheets were successfully fabricated through patterned electrodes, in which the electric field distribution and sheet alignment well-followed the electrode configuration. Finally, the aligned MXene film showed an anisotropic electrical property. The resultant conductivity along the basal plane (parallel direction) of the aligned MXene sheets is over 8 times higher than that of through the planes (orthogonal direction). This study can suggest a facile method to vertically align MXene sheets, enabling possibilities in future electrical, optical, or electrochemical applications.

## Methods

### Synthesis of Ti_3_C_2_T_*x*_ MXene

Ti_3_C_2_T_*x*_ MXene is synthesized using the MILD etching route by etching Ti_3_AlC_2_ MAX phases (Carbon, Ukraine)^54^. First, 9 M HCl (20 mL, Daejung Chemicals) was prepared in a polypropylene bottle. Then, LiF (1.6 g, Alfa Aesar) was added to the bottle is stirred at 320 rpm at 35 °C for at least 5 min for complete dissolution. While continuously stirring, Ti_3_AlC_2_ powder (1 g) is slowly added to the solution and is left to react for 24 h. After the reaction is complete, the solution is transferred to a Falcon tube and is centrifuged at 6500 x g for 5 min. Then, the supernatant is discarded, DI water is added to the tube, and the solution is centrifuged under the same condition. This process is repeated until the pH of the solution is close to 6. After discarding the supernatant at pH = 6, water is added to the sediment, and the tube is subjected to vigorous hand-shaking for 10 min to delaminate the MXene sheets. During this step, the amount of water added is controlled depending on the desired concentration of the final MXene solution. Then, the resulting solution is centrifuged at 2540 x g for 30 min to settle non-etched MAX crystals and non-delaminated MXene sheets. Finally, the supernatant containing delaminated Ti_3_C_2_T_*x*_ MXene sheets is collected for further use.

### ITO electrode patterning

ITO is sputtered on the glass substrate to be 170 nm. 1 µm of positive photoresist (AZ GXR 601, NM Tech) is coated on the ITO film, and UV is exposed through the designed mask. The irradiated region is removed using a developer (AZ MIF 300, NM Tech), and etching is followed in FeCl_3_ solution (Sigma Aldrich). Then the residual photoresist is removed.

### MXene cell preparation

A cell for applying an AC electric field to the MXene sample is prepared to assemble an electrode cell and a glass substrate. First, the ITO electrode is patterned on a 2.5 cm * 2.5 cm glass substrate to be a bottom substrate. 100 μm-thick silicon rubber sheet is placed on the bottom substrate without wrinkling. The sheet is well fixed to the substrate and serves as a spacer between the top and bottom substrates. An empty window of 1 cm by 1 cm is made by cutting out the sheet on the bottom substrate. 20 uL of MXene sample is dropped into the window on the bottom substrate. Finally, the top substrate, which is an ordinary glass, is covered over it and then gently pressed for perfect contact with the silicon rubber spacer to prevent solvent evaporation as much as possible. An in-plane electric field is applied to the MXene sample in the enclosed cell to induce vertical alignment.

### Freeze-drying

With an electric field applied, the MXene cell is carefully immersed in liquid nitrogen, keeping it as horizontal as possible to avoid temperature gradients within the sample. Initial freezing under an electric field is performed in liquid nitrogen for 30 min. Post-freezing is maintained for 2 h with the electric field turned off. The perfectly frozen sample maintaining the alignment is placed in a freeze-dryer chamber (FD8508, IlShin Biobase) and dried under vacuum for 24 h.

### Characterisation

The optical textures of the MXene sample were observed using POM (LV100POL, Nikon) with a CCD camera (DS-Ri1, Nikon). Scanning electron microscopy (SEM) (SU8230, Hitachi) was used to observe the morphology of the freeze-dried MXene sheets. Electrical property was determined by obtaining an I-V curve via voltage sweep from −1 V to 1 V between the two terminals using a semi-auto probe station (MPS20, Keysight).

### Reporting summary

Further information on research design is available in the Nature Research Reporting Summary linked to this article.

## Supplementary information

Supplementary Information

Description of Additional Supplementary Files

Supplementary Movie 1

Reporting Summary

## Data Availability

Source data are provided with this paper. All data generated in this study are provided in the main text, Supplementary Information and Source Data file.

## Source data

Source Data
