# Supplementary material for: Field-induced orientational switching produces vertically aligned Ti3C2Tx MXene nanosheets
Source: Nat Commun. 2022 Sep 24;13:5615. doi: 10.1038/s41467-022-33337-2 (PMC9509325; doi:10.1038/s41467-022-33337-2)
Supplement: Supplementary file 1 — Supplementary Information [file 41467_2022_33337_MOESM1_ESM.pdf]

Supplementary Information

Field-induced orientational switching produces vertically aligned Ti3C2Tx MXene nanosheets

Changjae Lee[1 †], Soon Mo Park[2 †], Soobin Kim[3, 4], Yun-Seok Choi[1], Geonhyeong Park[1], Yun Chan Kang[4], Chong Min Koo[3, 5, 6], Seon Joon Kim[3, 5, 7], Dong Ki Yoon[1, 2]**

[1] Department of Chemistry, Korea Advanced Institute of Science and Technology, Daejeon, 34141, Republic of Korea

[2] Graduate School of Nanoscience and Technology, Korea Advanced Institute of Science and Technology, Daejeon, 34141, Republic of Korea

[3] Materials Architecturing Research Center, Korea Institute of Science and Technology, 5, Hwarang-ro 14-gil, Seongbuk-gu, Seoul 02792, Republic of Korea

[4] Department of Materials Science and Engineering, Korea University, 145, Anam-ro, Seongbuk-gu, Seoul 02841, Republic of Korea

[5] Division of Nanoscience and Technology, KIST School, University of Science and Technology, 5, Hwarang-ro 14-gil, Seongbuk-gu, Seoul 02792, Republic of Korea

[6] School of Advanced Materials Science and Engineering, Sungkyunkwan University, Suwon 16419, Republic of Korea

[7]Convergence Research Center for Solutions to Electromagnetic Interference in Future-mobility, Korea Institute of Science and Technology, 5, Hwarang-ro 14-gil, Seongbuk-gu, Seoul 02792, Republic of Korea

†These authors contributed equally to this work.

*To whom correspondence should be addressed.

E-mail: seonjkim@kist.re.kr (S.J.K.), nandk@kaist.ac.kr (D.K.Y.)

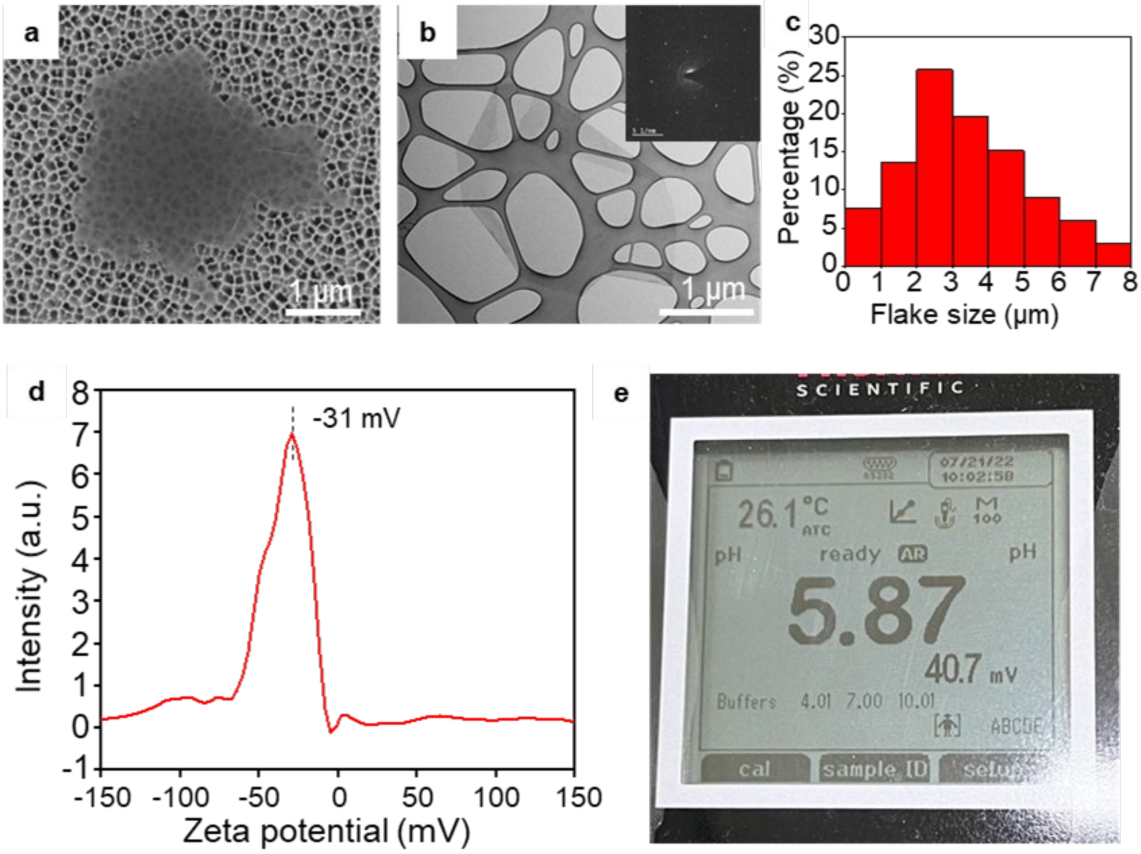

Supplementary Fig. S1. a, SEM and **b,** TEM image of a single delaminated MXene flake. The inset of b shows the SAED pattern. **c,** Flake size distribution of MXene flakes. **d**, Zeta-potential of MXene sheets and **e**, pH value of the MXene aqueous solution. Source data are provided as a Source Data file.

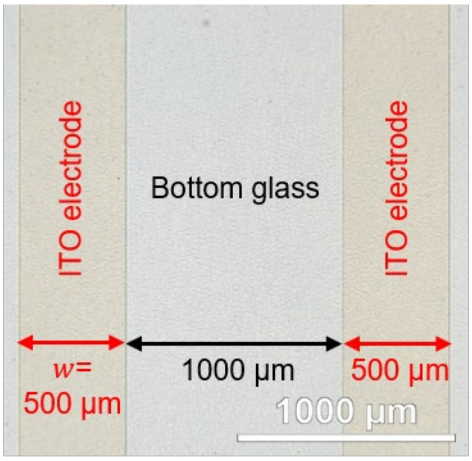

Supplementary Fig. S2. OM image of electrode substrate.

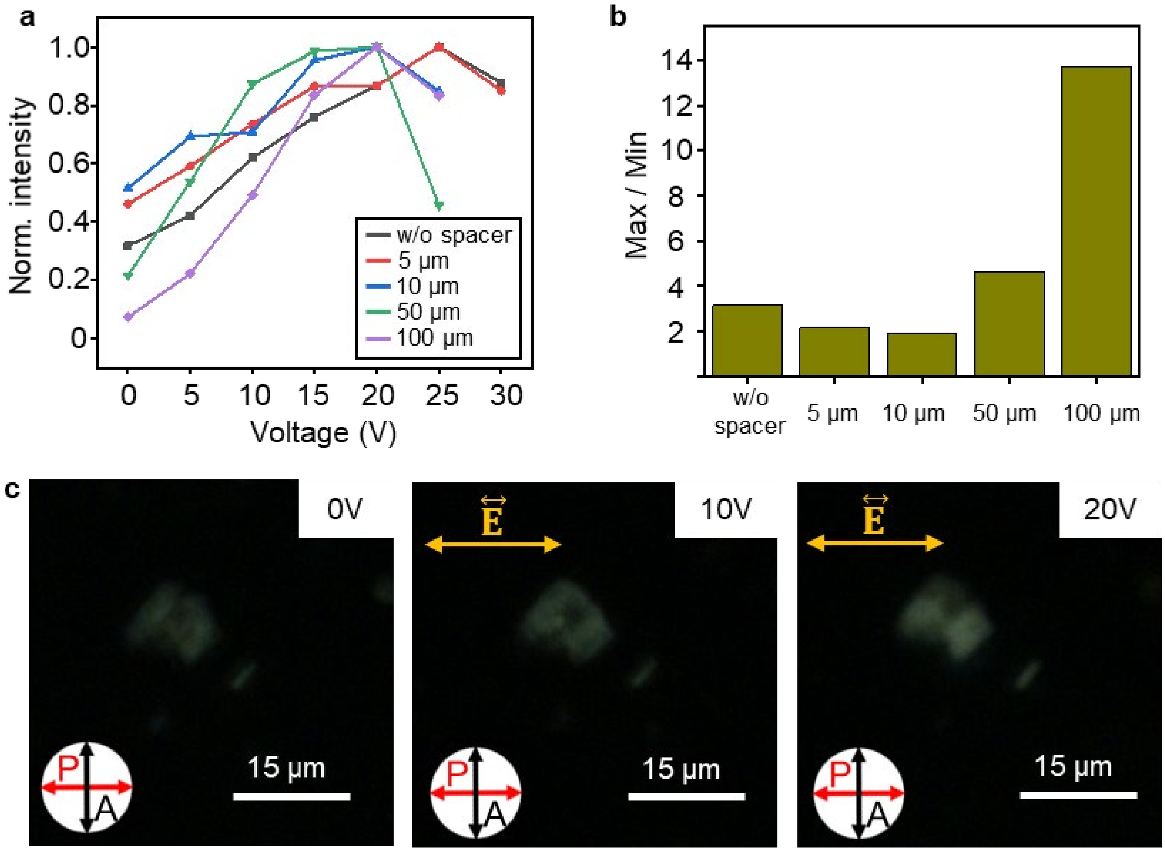

Supplementary Fig. S3. Thickness variation vs. birefringence. **a,** Changes in normalized intensity in each thickness as varying applied voltages. The intensity is normalized by the maximum intensity in each case. **b,** Comparison of ratios of maximum and minimum of the intensity. **c,** Large particle behaviour under an electric field. Source data are provided as a Source Data file.

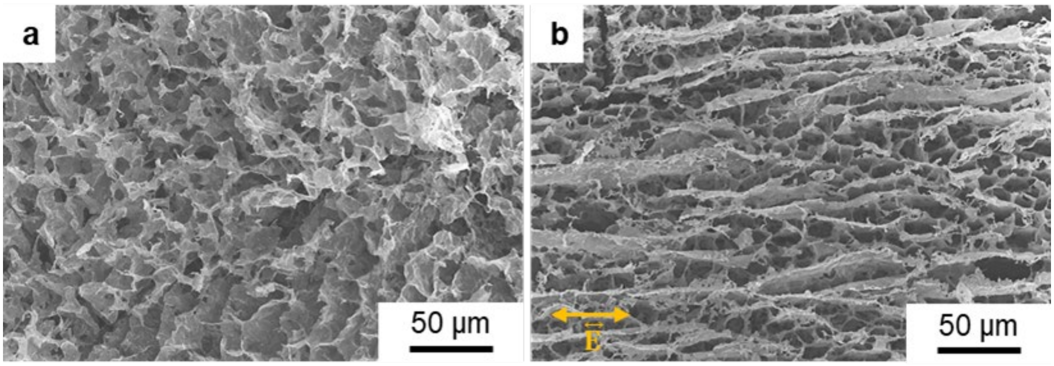

Supplementary Fig. S4. Top-view SEM image of freeze-dried MXene sheets prepared without (**a**) and with (**b**) an electric field.

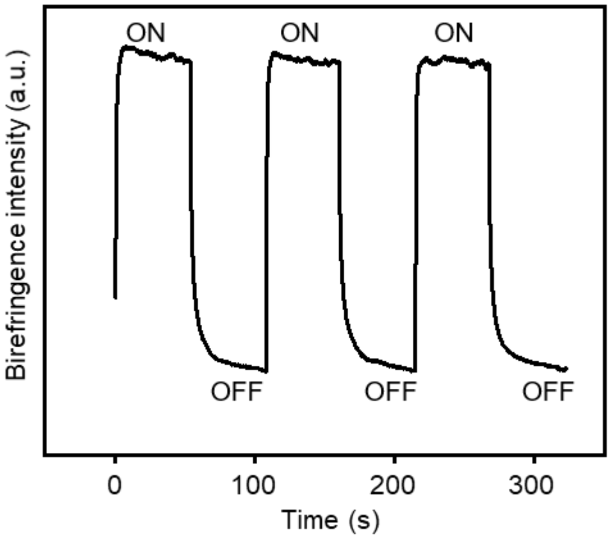

Supplementary Fig. S5. Reversibility test depending on the applied and relaxation time. Source data are provided as a Source Data file.

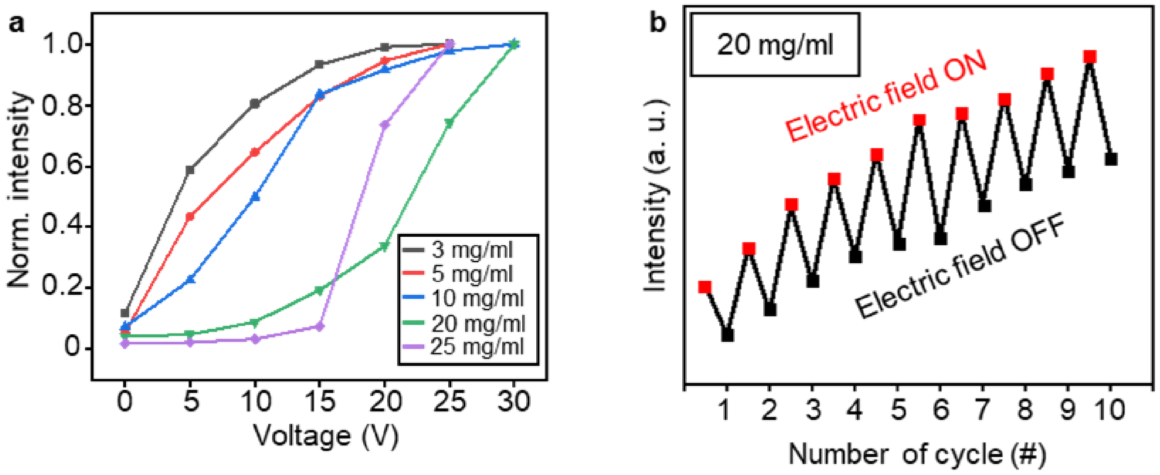

Supplementary Fig. S6. Concentration variation. a, Birefringence change of varied concentration increasing applied voltage. The intensity is normalized by the maximum intensity in each case. **b,** Reversibility test of 20 mg/ml MXene solution. Source data are provided as a Source Data file.

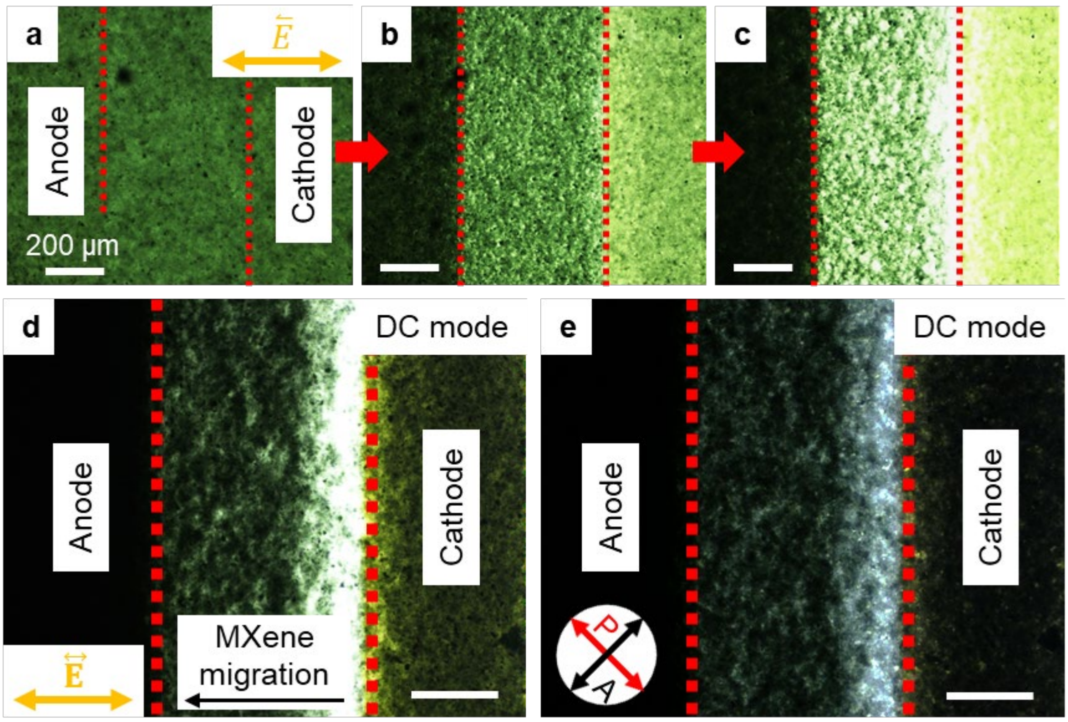

Supplementary Fig. S7. Current mode-dependent optical texture of MXene liquid crystal. **a-c,** OM image under the application of a DC electric field over time. **d-e,** Final images under DC filed with a single polariser (**d**) and crossed polarisers (**e**).

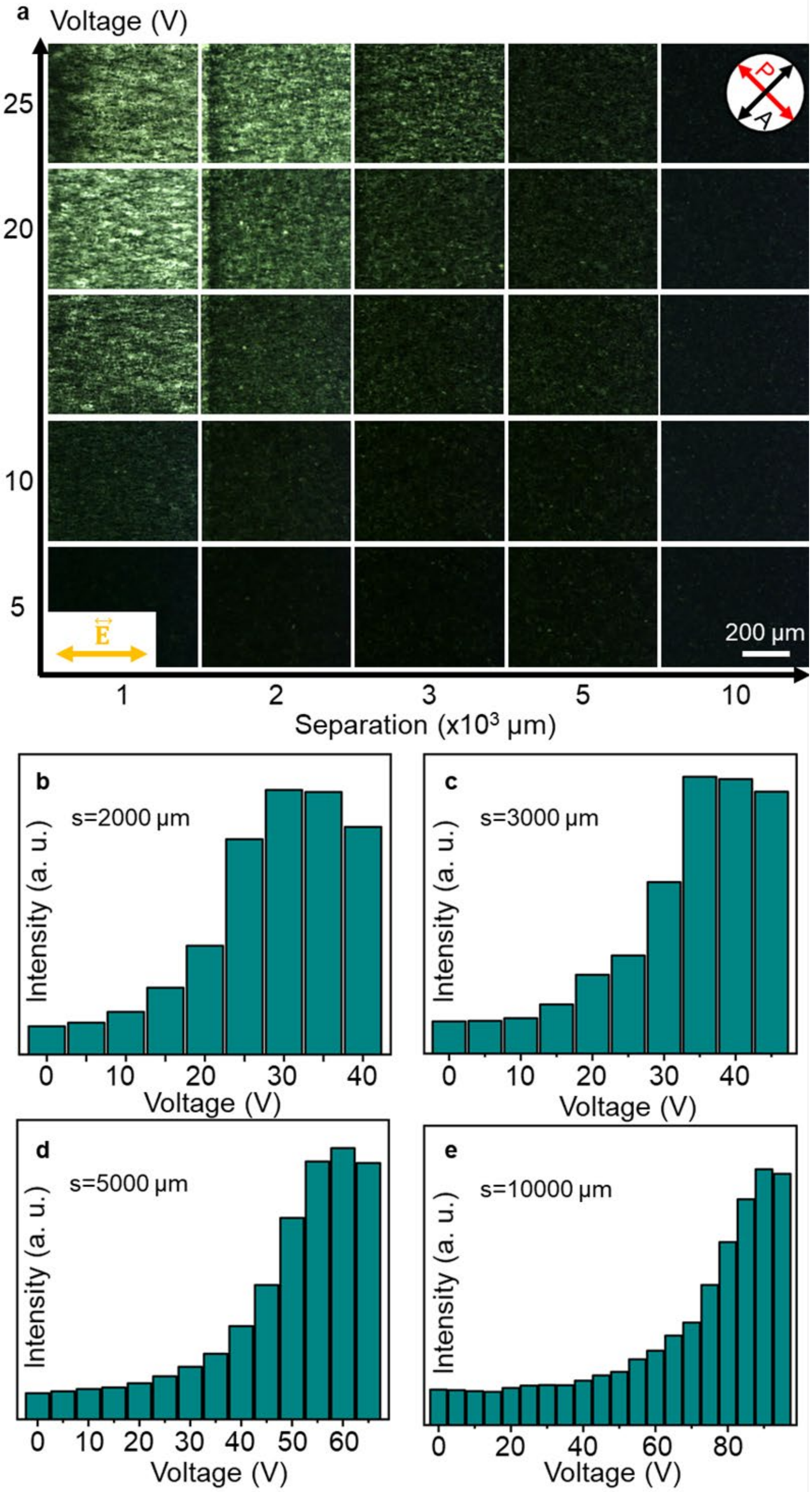

a
Voltage (V)
25
20
10
5
P
A
E
200 µm
1
2
3
5
10
Separation (x10³ µm)
b
s=2000 µm
Intensity (a. u.)
0
10
20
30
40
Voltage (V)
c
s=3000 µm
Intensity (a. u.)
0
10
20
30
40
Voltage (V)
d
s=5000 µm
Intensity (a. u.)
0
10
20
30
40
50
60
Voltage (V)
e
s=10000 µm
Intensity (a. u.)
0
20
40
60
80
Voltage (V)

Supplementary Fig. S8. **a,** POM images varying the voltages and electrode separations. **b-e,** Birefringent intensity of each electrode separation depending on the applied voltage. Source data are provided as a Source Data file.

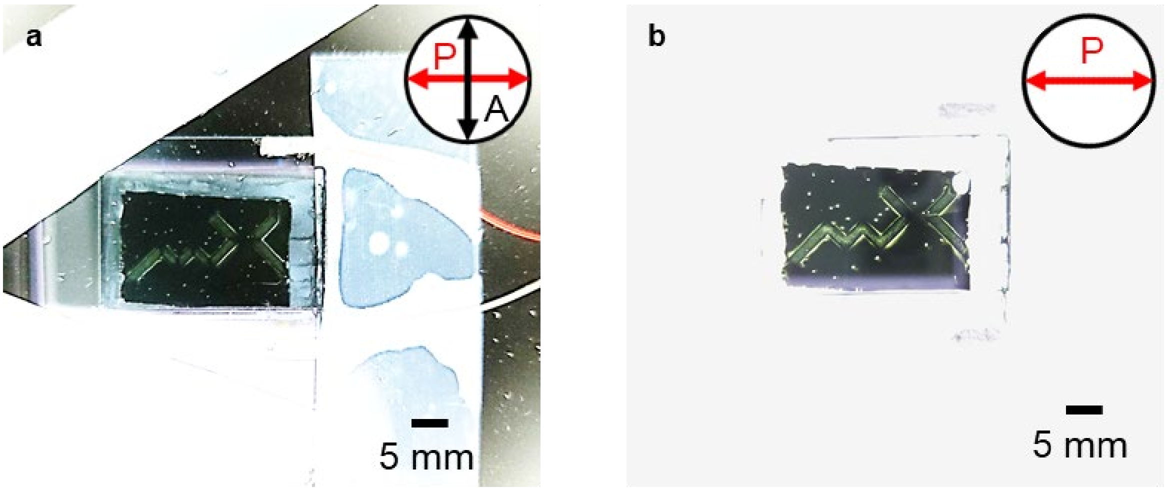

Supplementary Fig. S9. Original images of MXene arrangement forming 'MX' pattern. a, Images with crossed polarisers. **b,** Photo with a single polariser

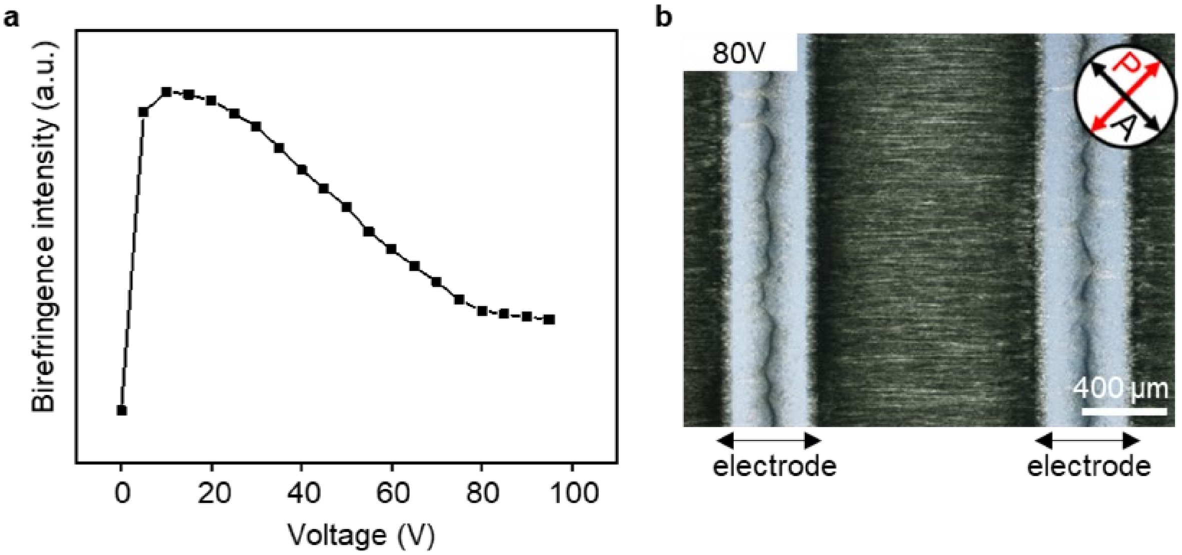

Supplementary Fig. S10. Dielectrophoretic condensation in DMSO MXene solution. a,

Change in birefringence intensity of DMSO MXene solution. **b,** POM image of DMSO MXene solution at 80 V. Source data are provided as a Source Data file.
