# Supplementary material for: Field-induced orientational switching produces vertically aligned Ti3C2Tx MXene nanosheets
Source: Nat Commun. 2022 Sep 24;13:5615. doi: 10.1038/s41467-022-33337-2 (PMC9509325; doi:10.1038/s41467-022-33337-2)
Supplement: Supplementary file 2 — Description of Additional Supplementary Files [file 41467_2022_33337_MOESM2_ESM.pdf]

Description of Additional Supplementary Files

File Name: Supplementary Movie 1

Description: Field-induced reversible switching of MXene solution
